# Cerebrovascular Accident due to Thyroid Storm: Should We Anticoagulate?

**DOI:** 10.1155/2016/5218985

**Published:** 2016-08-15

**Authors:** Alex Gonzalez-Bossolo, Alexis Gonzalez-Rivera, Santiago Coste-Sibilia

**Affiliations:** Internal Medicine Training Program, Department of Medicine, University District Hospital, University of Puerto Rico School of Medicine, P.O. Box 365067, San Juan, PR 00936-5067, USA

## Abstract

Thyroid storm is a life-threatening condition that occurs secondary to an uncontrolled hyperthyroid state. Atrial fibrillation is a cardiovascular complication occurring in up to 15% of patients experiencing thyroid storm, and if left untreated this condition could have up to a 25% mortality rate. Thyroid storm with stroke is a rare presentation. This case report details a left middle cerebral artery (MCA) stroke with global aphasia and thyroid storm in a 53-year-old Hispanic male patient. Although uncommon, this combination has been reported in multiple case series. Although it is well documented that dysfunctional thyroid levels promote a hypercoagulable state, available guidelines from multiple entities are unclear on whether anticoagulation therapy is appropriate in this situation.

## 1. Introduction

Thyroid storm is a life-threatening condition that is associated with an uncontrolled hyperthyroid state caused by a precipitating event, such as recent surgery, trauma, infection, iodine load, or poor adherence to antithyroid medications [1]. Although tachycardia is the most common cardiovascular manifestation of thyroid storm, atrial fibrillation occurs in up to 15% of patients [2, 3]. Thyroid hormone dysregulation affects the coagulation pathway and promotes settings in which a stroke may occur [4]. There is, however, no clear recommendation for anticoagulation therapy in patients experiencing thyroid storm. This case report describes a 53-year-old Hispanic man who experienced a left middle cerebral artery (MCA) stroke and global aphasia with coexisting thyroid storm.

## 2. Case Presentation

**Figure 1:**
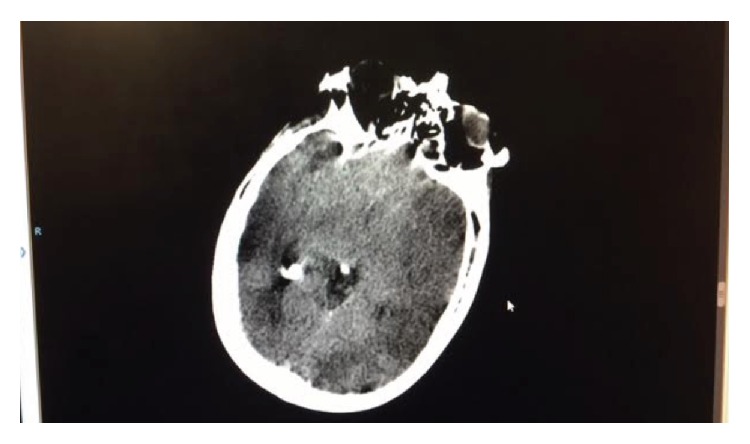
Head computed tomography image without contrast, showing left middle cerebral artery territory infarction.

**Figure 2:**
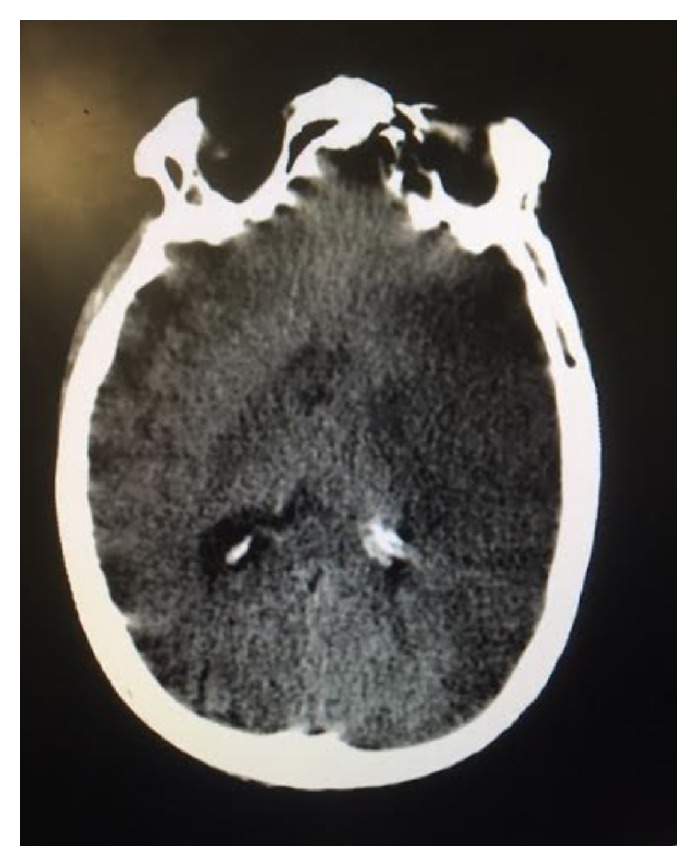
High density within left middle cerebral artery territory, corresponding to the site of arterial occlusion.

A 53-year-old man with a history of untreated hyperthyroidism (toxic nodule), chronic smoking, and arterial hypertension arrived to the emergency room of our institution complaining of disorientation, restlessness, palpitations, vomiting, abdominal pain, and fever (not quantified) that had begun over 48 hours prior. Worsening symptoms, including right-sided weakness and difficulty understanding verbal language and communication with his spouse in the previous 24 hours, prompted the patient's assistance to our institution. Upon evaluation by the emergency room physician, the patient had a decreased neurological status with poor response to pain and verbal stimuli, for which he was endotracheally intubated to secure his airway. A head computed tomography (CT) scan without intravenous contrast revealed a gross acute left middle cerebral artery (MCA) territory infarction with no associated hemorrhage and minimal compression of the left lateral ventricle (Figures 1 and 2). The patient had a National Institutes of Health stroke scale value of 9/42, so the emergency department consulted the internal medicine department for further management. Thrombolytic therapy was not indicated due to the timing of symptom appearance.

Upon evaluation in our department, we determined that the patient was acutely ill with a Glasgow Coma Scale score 11/15 when assessed while off sedation and on mechanical ventilation support with adequate oxygenation. His vital signs showed that he had a remarkable high fever (39.8°C), tachycardia (heart rate: 107 bpm), and elevated blood pressure 180/90 mmHg. A physical examination revealed right hemiplegia with bibasilar crackles and an irregular rhythm. He also exhibited remarkable pedal edema. An electrocardiogram revealed atrial fibrillation with adequate ventricular response. Due to a patient history of untreated hyperthyroidism secondary to problems with medical insurance as well as clinical signs and symptoms, the patient was diagnosed with thyroid storm. The patient had a Burch and Wartofsky score of 70, which is indicative of thyroid storm [5]. Treatment was initiated immediately, using a beta blocker (60 mg of propranolol) to control adrenergic symptoms, a thionamide (200 mg of propylthiouracil) to block hormone synthesis, and a glucocorticoid (100 mg of hydrocortisone) to reduce T4-T3 conversion. Lugol's iodine solution (10 drops of 8 mg iodine per 0.05 mL drop) used to stop thyroid hormone synthesis was initiated 1 hour after the antithyroid drugs. Additionally, along with statin therapy (atorvastatin 80 mg), clopidogrel 75 mg and enoxaparin 40 mg were administered as secondary stroke prevention. Intravenous diuretics were given to treat fluid overload symptoms.

Laboratory results revealed no leukocytosis or leukopenia, adequate hemoglobin and hematocrit levels, no electrolytes disturbances, and a negative toxicology screen. Thyroid function tests were abnormal (thyroid stimulating hormone: <0.225 *µ*IU/mL, total thyroxine: 16.50 mcg/dL, and free thyroxine: 5.49 ng/dL) (Table 1). A chest radiograph revealed opacity projecting at the right lower lung field with silhouetting of the diaphragm and increased interstitial lung markings, which were consistent with pulmonary edema. A two-dimensional echocardiogram revealed atrial fibrillation at baseline, mild left ventricular systolic dysfunction, no valvulopathies, and mild left atrium and right atrium dilation. Based on the Japanese Thyroid Association criteria for thyroid storm, this case met the criteria for definitive diagnosis of TS1-grade thyroid storm [5].

Our patient was treated aggressively for thyroid storm upon arrival to our institution. Successful extubation was achieve on day 6 following admission. When the patient's mental status recovered, he confirmed that his fever palpitations and nausea preceded the paralysis, confirming that the events of thyrotoxicosis leaded to the stroke. In addition, his sinus rhythm returned to normal after completed hyperthyroidism treatment and his pedal edema improved. The patient was transferred to a rehabilitation center for inpatient physical therapy on the 8th day of admission. Following physical therapy sessions, he was discharged and given thionamide therapy with methimazole for his hyperthyroidism and was followed up by primary endocrinologist for further management. Based on the clinical progression of the patient's illness, we feel that if early anticoagulation had been started, the cerebrovascular accident with associated global aphasia might have been prevented.

## 3. Discussion

Thyroid storm is a life-threatening condition that occurs due to an accentuated hyperthyroid state. Recent studies report a mortality range from 8 to 25% [6]. Thyroid storm usually presents in patients with a current history of poorly treated thyrotoxicosis. It can also be precipitated by an infection, recent surgery, or recent trauma, among other things. The clinical presentation resembles an accentuated catabolic state. Fever is a very common sign and is often accompanied by sweating. Other manifestations include agitation, diarrhea, nausea, vomiting, abdominal pain, jaundice, seizures, and coma [5, 7]. A link between the cardiovascular system and elevated thyroid function has also been characterized. Sinus tachycardia is the most common electrocardiographic finding, but atrial fibrillation can also occur and is the most common cardiovascular complication, arising in 5–15% of cases [2, 3]. Another important link that was recently discovered is between thyroid hormone and coagulation pathways. Thyrotoxicosis promotes a hypercoagulable state due to a shortened activated partial thromboplastin time, increased fibrinogen levels, and increased factor VIII and factor X activity [4]. These abnormalities predispose a patient to stroke regardless of heart rhythm.

Although these links are known and the mechanisms have been documented, there are no specific or clear-cut recommendations regarding anticoagulation therapy in these patients. Guidelines from multiple entities also do not include hyperthyroidism as a risk factor for stroke. The most recent American Thyroid Association (ATA) guidelines for thyrotoxicosis management do not clearly state that anticoagulation therapy should be included only suggesting its use in the event of heart failure [8]. In 2014, the American Heart Association (AHA), American College Cardiology (ACC), and the Heart Rhythm Society (HRS) published guidelines for atrial fibrillation (AF) management. They stated that the evidence associating thyrotoxicosis with AF was not sufficient enough to recommend anticoagulation therapy administration. Anticoagulation should be guided by the CHA2DS2-VASc risk factors, which do not include hyperthyroidism [9]. Although no large randomized controlled trials have assessed this association, many case reports/series have documented it. Yuen et al. reported a case series of 21 subjects with thyrotoxicosis and atrial fibrillation, in which 23% of the patients had developed systemic emboli [10]. In another study, Bar-Sela and associates describe 30 out of 142 thyrotoxic patients with concomitant atrial fibrillation, 12 (40%) of whom experienced an embolic event [11]. In a recent prospective cohort study from Taiwan, 3176 patients with hyperthyroidism were followed for 5 years and compared with subjects without hyperthyroidism. The risk for developing a stroke was 1.4-fold greater in the hyperthyroidism cohort compared with the control group after adjusting for several confounders [12].

In conclusion, although some clinical evidence exists concerning cardioembolic stroke in thyrotoxic patients, no standard of care exists in this situation, likely because the clinical protocol is based on case reports and methodologically flawed small-scale studies. However, the decision for anticoagulation in thyrotoxicosis should be based on the patient's risk factors, as shown in previous studies, and not solely on the presence of thyroid storm. The case presented here highlights an important unresolved issue regarding the administration of anticoagulation therapy in patients with thyrotoxicosis with atrial fibrillation.

## Figures and Tables

**Table 1:** Thyroid function tests and serum chemistries.

	Upon hospital arrival	Upon discharge
Thyroid stimulating hormone (*µ*IU/mL)	<0.225	N/A^a^
Total T4 (*µ*g/dL)	16.50	12.2
Free T4 (ng/dL)	5.49	2.3
Sodium (mEq/L)	147	143
Potassium (mEq/L)	3.8	3.5
Blood urea nitrogen (mg/dL)	32	29.5
Creatinine (mg/dL)	0.82	0.50
Calcium (mg/dL)	8.5	8.4
Phosphorus (mg/dL)	3.70	3.5
Magnesium (mg/dL)	2.22	2.0
Albumin (g/dL)	2.7	2.5
Carbon dioxide (mEq/L)	25.2	26.7
Prothrombin time (s)	13	N/A^a^
Partial thromboplastin time (s)	30	N/A^a^
INR (s)	1.0	N/A^a^
White blood cell (×10^3^/*μ*L]	8.8	7.5
Hemoglobin (g/dL)	14.7	14.0
Hematocrit (%)	44	42
Platelet count (×10^9^/L)	300	350

^a^Value not available.
